# 

**Published:** 1877-06-20

**Authors:** 


ORIGINAL AND SELECTED ARTICLES—

Sulphate of Cinchonidia and Sulphate of Quinine. By Prof. Wm. Abram Love, M. D. ..........139

Glycerite of Kephaline. By C. G. Polk, M.D ..........1

Aconite. By I. J. M. Goss, M. D. ..........143

Remedies for Cough ..........146

On the Best Means of Promoting Union by First Intention. By E. W. Lee, M. D. ..........148

ABSTRACTS AND GLEANINGS—

Elixir Glycyrrhizæ, 150. Cancer, 151. Bandaging for Ulcers on the Leg, 151. Apocynanum Canabinum in Dropsical Affections, 151. On the Hypothesis that the Human Ovaries contain Male or Female Ova, 151. Dioscoria Villosa, or Wild Yam, 152. Cocoa, 152. Chronic Chills, 152 Mixture for Nervous Insomnia, 153. Effects of Medicine upon the Fetus in Utero, 153. Diphtheria, 153 Progressive Pernicious Anæmia Treated by Transfusion, 154. Croton Chloral in Toothache, 154. Glycerine a Poison, 157 Fucus Vesiculosus, 154. Laparotomy, 155. Sick Stomach, 155. A New Treatment in Post-partum Hemorrhage, 156. Sweet Spirits of Nitre a Solvent for Salicylic Acid, 156. Hydrocele, 156. Sulphurous Acid in Chronic Urticaria, 156.

PRACTICAL NOTES—

Cholera Infantum, 157. Dysentery, 158. Ipecac Treatment of Dysentery, 159. Corneal Opacities, 159. Corneal Ulcer, 159 Ascarides Vermicularis, 159. Chloral Locally in Neuralgia, 159. Apthous Ulcerations, 160. Kerosene Oil in Croup, 160. Chloral for False Membrane, 160. Jaundice, 160 Lumbrocoides, 160. Sulphate of Zinc in Chorea, 160. Tooth Powder, 160. Chronic Gleet, 160. Caution in the Use of Salacylic Acid, 160 Chronic Hepatitis, 161. Acid Deposits in the Urine—Gravel, 161. Chronic Mammitis, 161. Hæmaturia, 161. Angina Pectoris, 162. Gallic Acid in Phthisis, 162. Chloroform as a Liniment 162. Acute Nephritis, 162 Milk Beer, 162. The Use of Hypophosphites of Lime and Soda in Phthisis, 162. Purgative and Diuretic, 163. Peritonitis, 163. Cholera Morbus, 163. Obstinate Diarrhœa, 163. Anæsthetic Mixtures, 163. Casca Bark, 163.

SCIENTIFIC ITEMS—

Materialistic Psychology, 163.

EDITORIAL AND MISCELLANEOUS—

Please Read, 164. The Pocket Case of Drugs, 164. American Medical Association, 165. Consultations—A Query, 166. University of New York, 166. Cinchonidia, 166.

